# Supplementary material for: Species invasiveness and community invasibility of North American freshwater fish fauna revealed via trait-based analysis
Source: Nat Commun. 2023 Apr 22;14:2332. doi: 10.1038/s41467-023-38107-2 (PMC10122662; doi:10.1038/s41467-023-38107-2)
Supplement: Supplementary file 1 — Supplementary Information [file 41467_2023_38107_MOESM1_ESM.pdf]

Supplementary Information for

Species invasiveness and community invasibility of North American freshwater fish fauna revealed via trait-based analysis

Guohuan Su[1 2*], Adam Mertel[1], Sébastien Brosse[3], Justin M. Calabrese[1,4,5]

[1]*Center for Advanced Systems Understanding (CASUS), Helmholtz-Zentrum Dresden-Rossendorf (HZDR), Görlitz, Germany*

[2] *Institute of Hydrobiology, Chinese Academy of Sciences, Wuhan, 430072, China*

[3]*Laboratoire Evolution et Diversité Biologique (EDB), Université de Toulouse, CNRS, IRD, UPS, Toulouse, France*

[4]*Department of Ecological Modelling, Helmholtz Centre for Environmental Research-UFZ, Leipzig, Germany*

[5]*Department of Biology, University of Maryland, College Park, MD, USA*

*Corresponding author. Email: guohuan.su@gmail.com

This PDF file includes:

Figs. S1 to S10

Tables S1 to S4

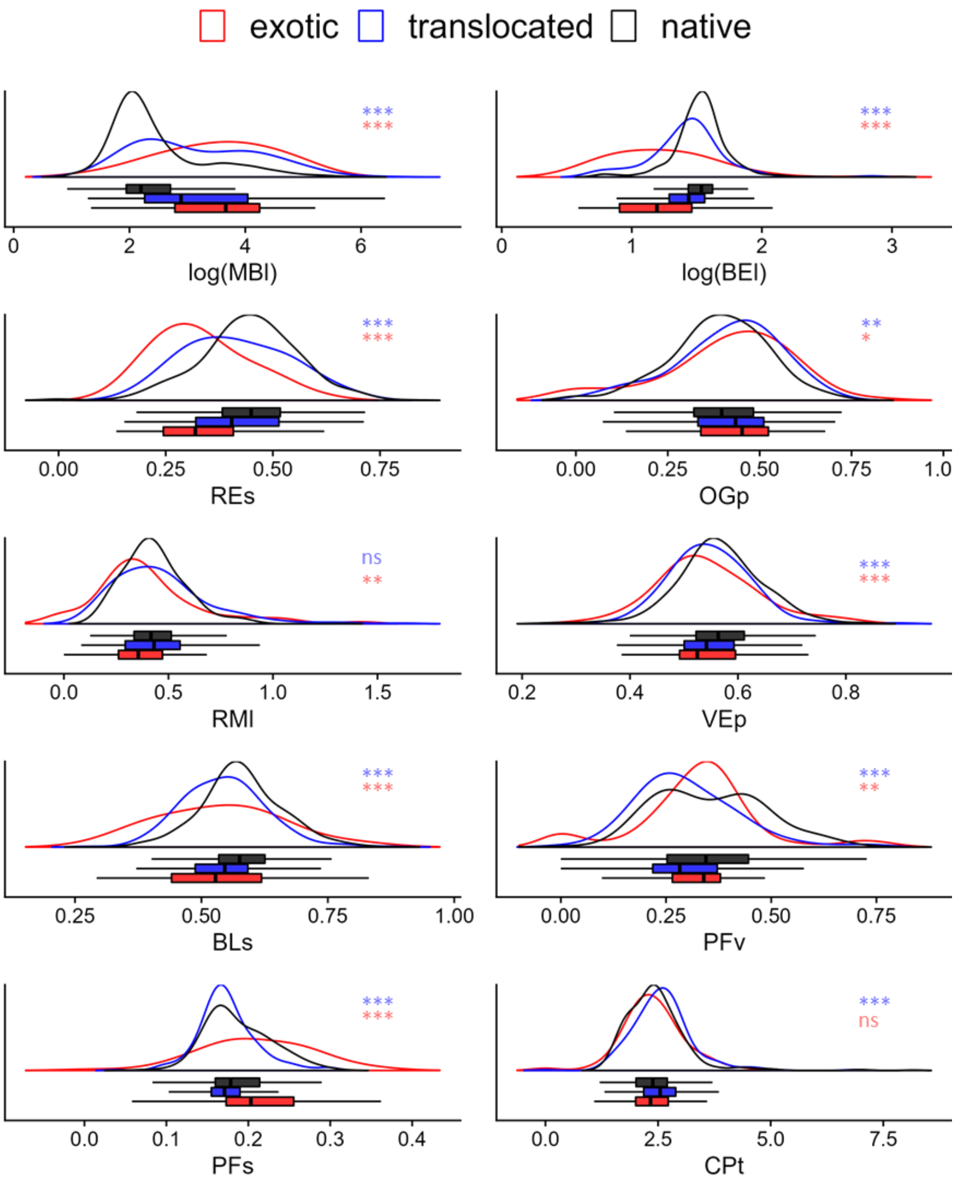

Fig. S1.

Density distribution of species on the 10 morphological trait axes for native, translocated and exotic species in the US. Boxplots and results of two-sided K-S tests of translocated-native (blue) and exotic-native (red) species are shown beside each plot item (*** $P < .001$, ** $P < .01$, * $P < .05$). Centre, bounds of box and whiskers of boxplots represent the median, 25% quantile,75% quantile, minima and maxima. quatSpecies number: native 559; translocated 227; exotic 73. See Table S2 for the complete results of K-S tests between the three groups. MBl: maximum body length; BEl: body elongation; REs: relative eye size; OGp: oral gape position; RMl: relative maxillary length; EVp: eye vertical position; BLs: body lateral shape; PFv: pectoral fin vertical position; PFs: Pectoral Fin Size; CPt: caudal peduncle throttling.

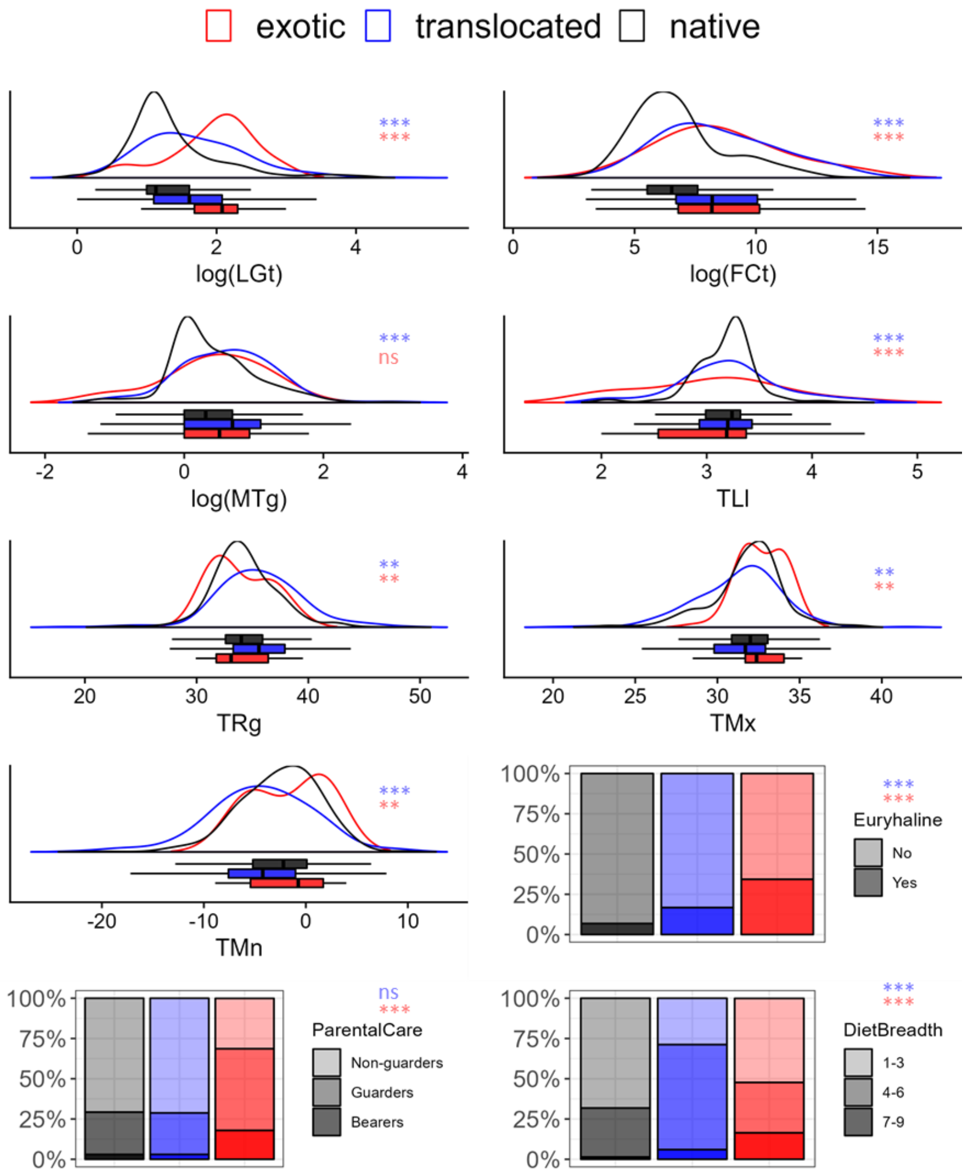

Fig. S2.

Density distribution of species on the other 10 life-historical trait axes for native, translocated and exotic species in US. Boxplots and results of two-sided K-S tests for the seven quantitative traits and two-sided Chi-square tests for the three categorical traits of translocated-native (blue) and exotic-native (red) species are shown beside each plot item (*** $P < .001$, ** $P < .01$, * $P < .05$). Centre, bounds of box and whiskers of boxplots represent the median, 25% quantile,75% quantile, minima and maxima. Species number: native 559; translocated 227; exotic 73. See Table S2 for the complete results of two-sided K-S tests for the first 7 quantitative traits and two-sided Chi-Square tests for the last 3 categorical traits between the three groups. LGt: longevity, FCt: fecundity; MTg: mature age; TLl: trophic level; TRg temperature range; TMx; maximum temperature; TMn: minimum temperature.

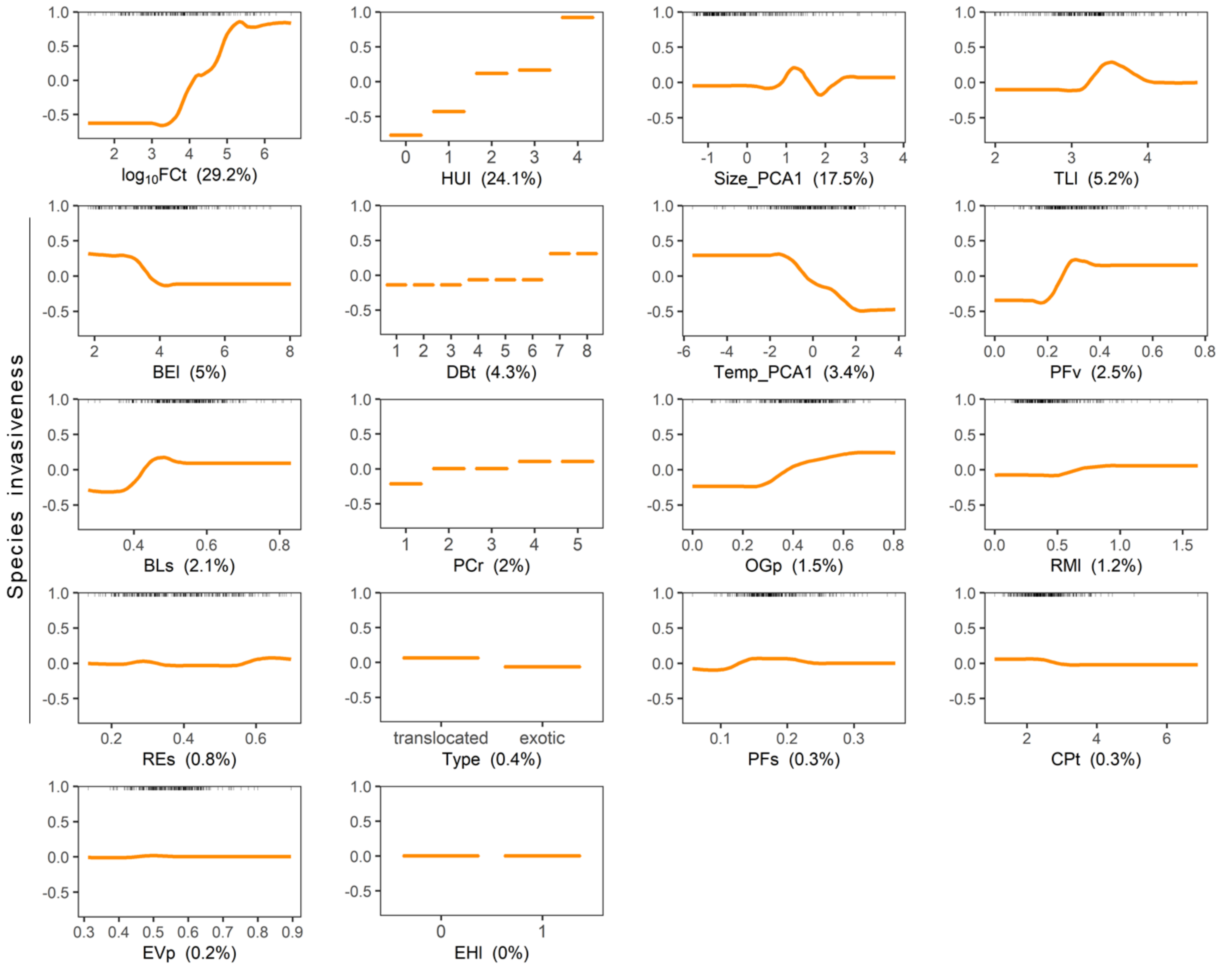

Fig. S3.

Results of boosted regression trees showing the partial dependency between species invasiveness and the 18 predictors. The value in parentheses in each panel shows the percentage of contribution of each trait considered in the model. The rugs in the top of each panel show the distribution of the species along the trait values. FCt: fecundity; HUI: human use index; Size_PCA1: first PCA axis of maximum body length, longevity, and mature age; TLl: trophic level; DBt: diet breadth; BEl: body elongation; Temp_PCA1: first PCA axis of temperature range, minimum and maximum values; BLs: body lateral shape; PFv: pectoral fin vertical position; PCr: parental care; RMl: relative maxillary length; REs: relative eye size; OGp: oral gape position; Type: translated or exotic; CPt: caudal peduncle throttling; PFs: pectoral fin size; EVp: eye vertical position; EHl: euryhaline.

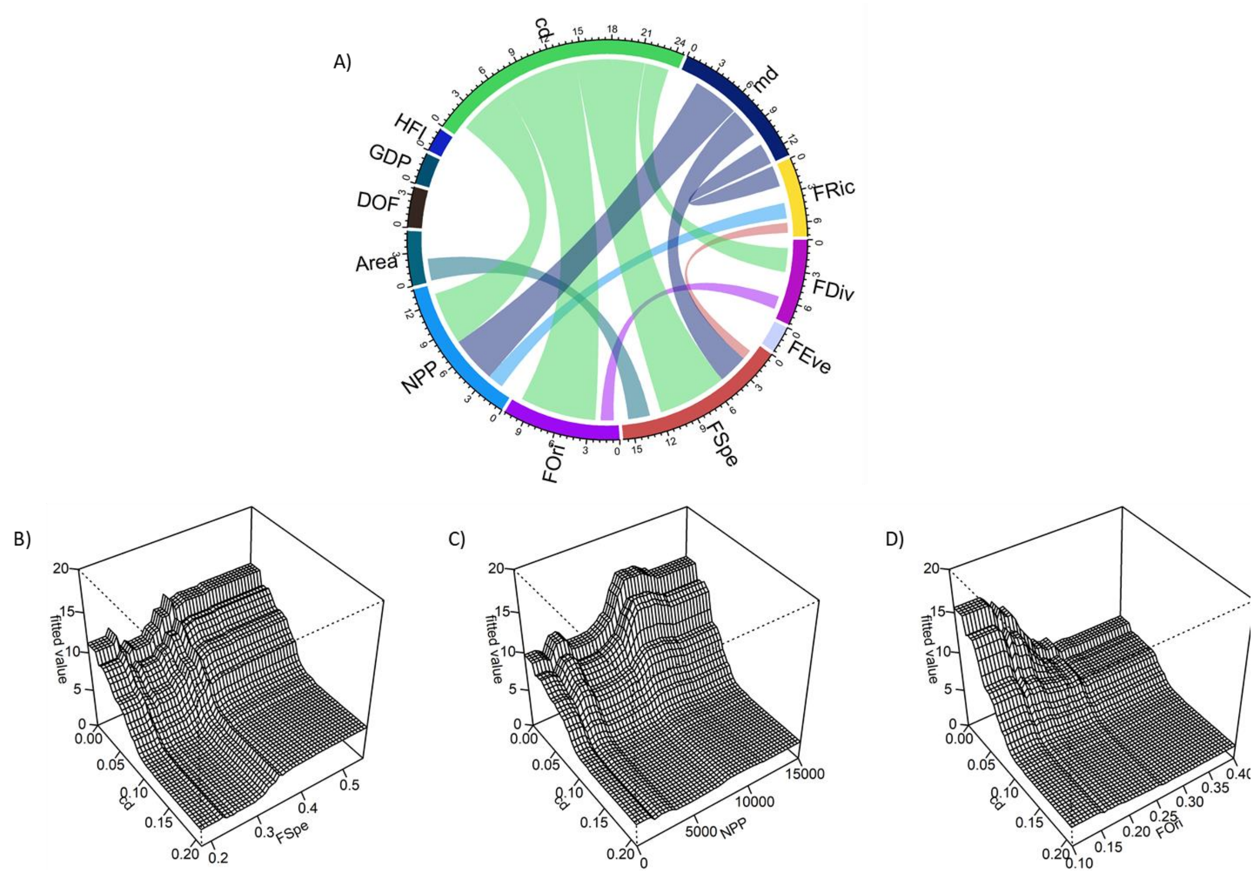

Fig. S4.

Interactions between the 12 predictors in the boosted regression tree (BRT) model for fish community invasibility. A) chordplot shows the main interactions (value > 1). The width of the bands represents the strength of interaction between pairs of the predictors used to build the BRT. B) to D) Three-dimensional surface plots showing fitted values as a function of the three pairs of strongest interacted predictors. FRic: functional richness; FDiv: functional divergence; FEve: functional evenness; FSpe: functional specialization; FOri: functional originality; DOF: degree of river fragmentation; NPP: net primary productivity; HFI: human footprint index; GDP: gross domestic product; Area: watershed area; cd: centroid distance; md: mean distance.

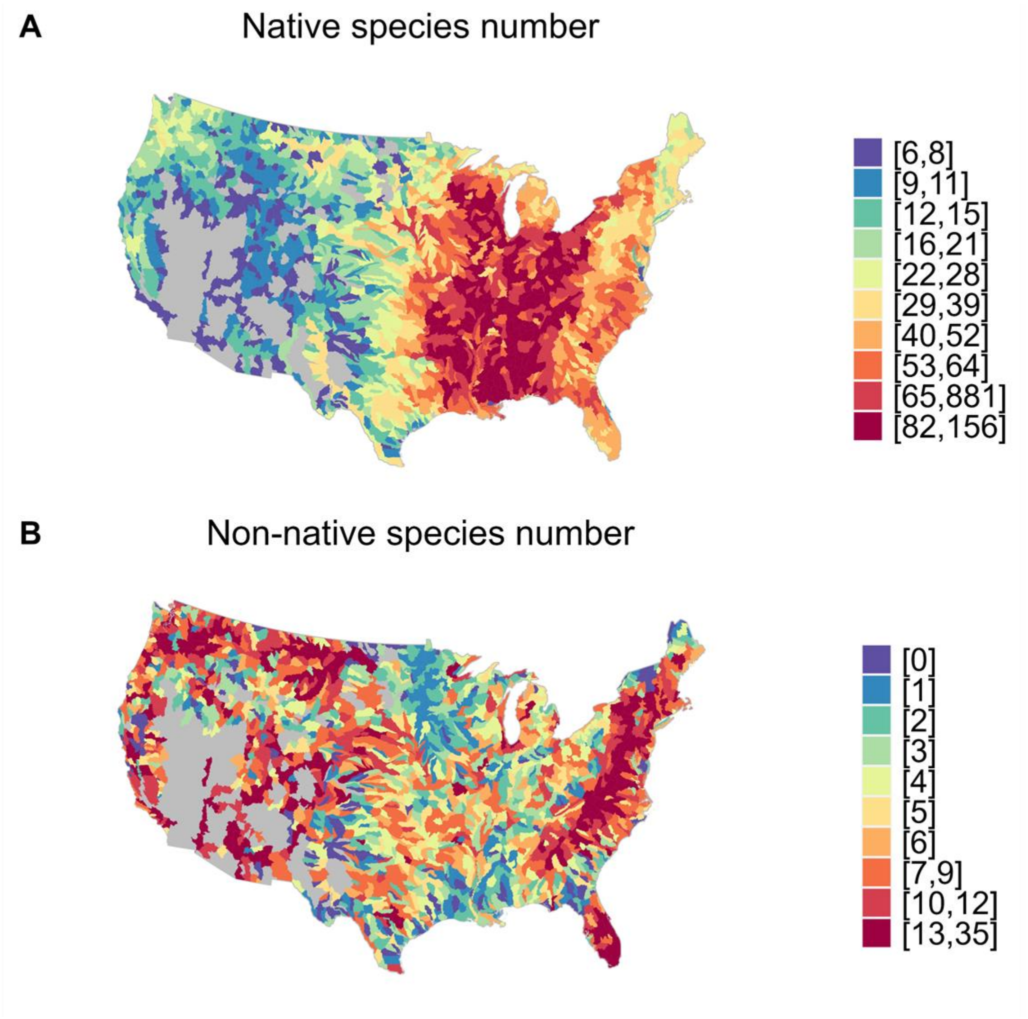

Fig. S5.
Pattern of native and non-native species in the 1,868 watersheds in the US.

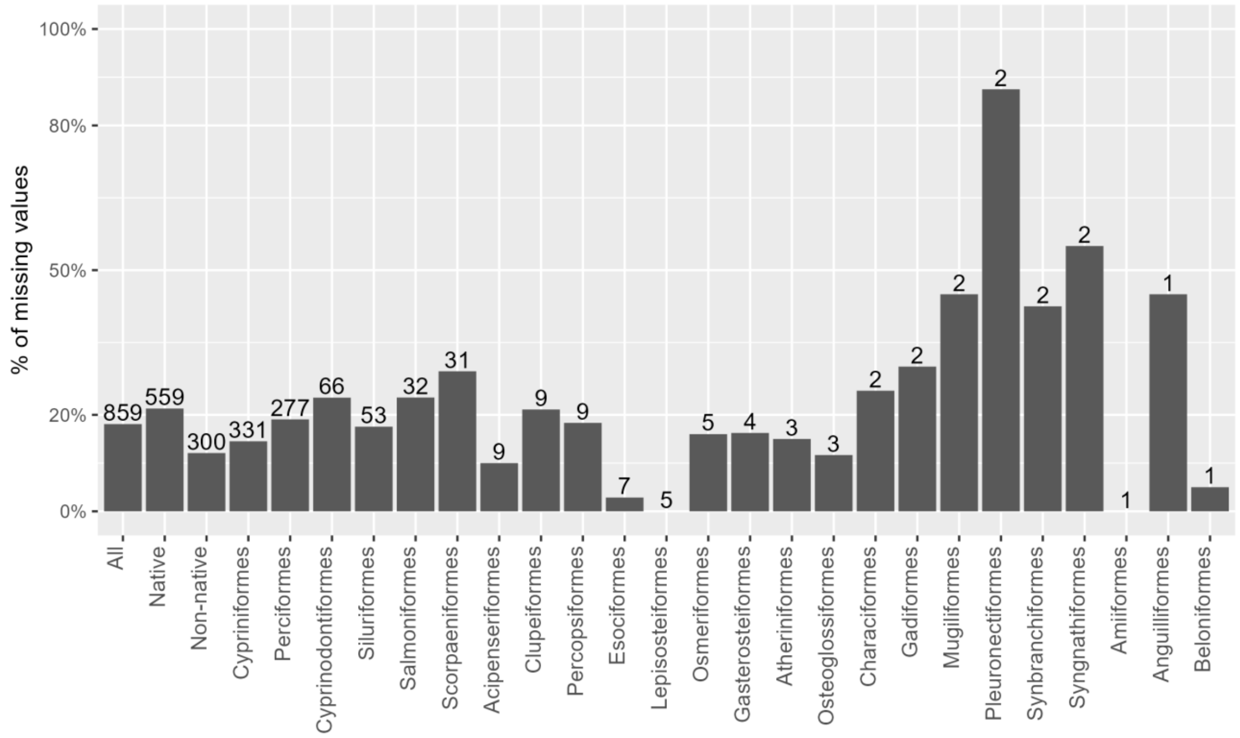

Fig. S6.

Distribution of missing values among the entire dataset, native (have never invaded other watersheds), non-native, and taxonomic orders. Values above the bars represent the numbers of species in the groups.

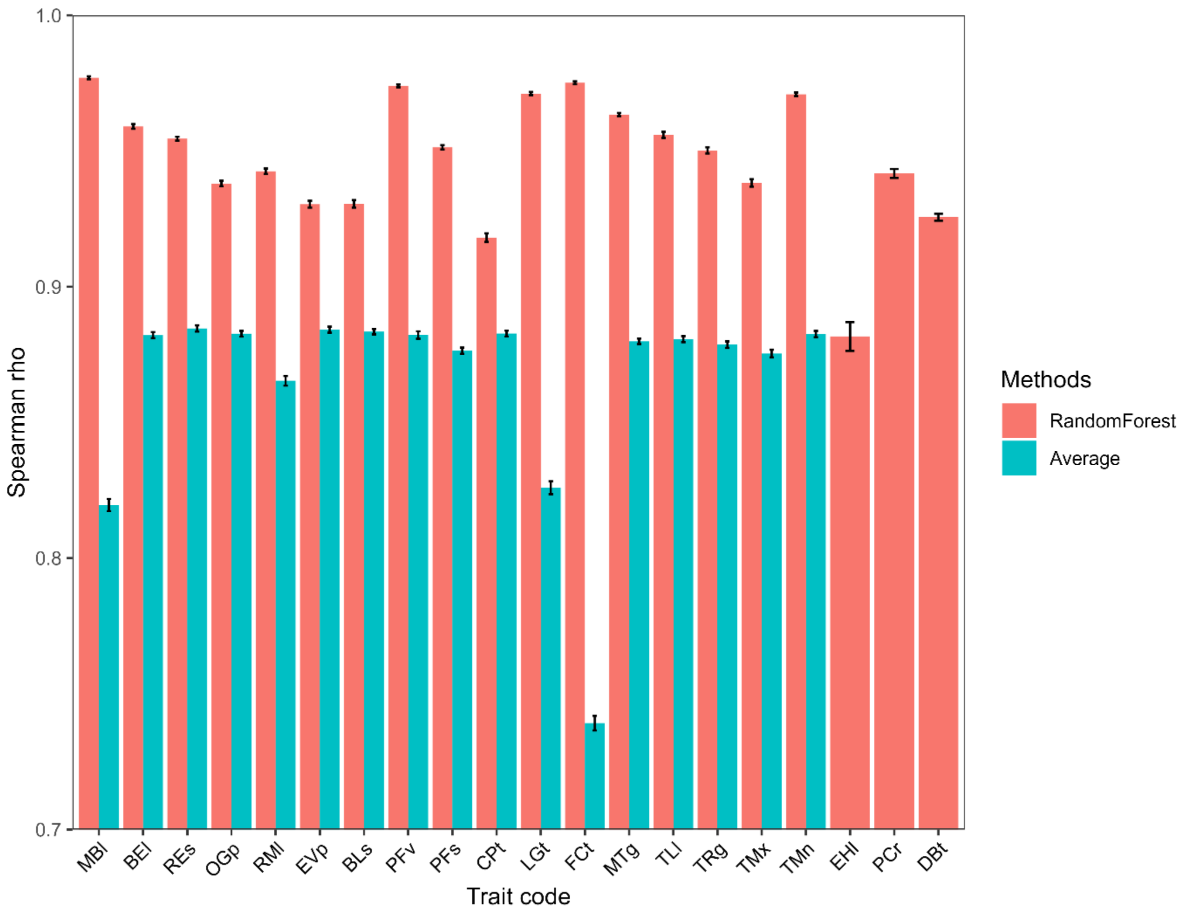

Fig. S7.

Comparison of the efficiency of filling the trait missing values using the random forest method and average values. Using average to fill missing values was conducted for the 17 continuous traits. Number of species used n = 350. Data are presented as mean values +/- SEM. MBl: maximum body length; BEl: body elongation; REs: relative eye size; OGp: oral gape position; RMl: relative maxillary length; EVp: eye vertical position; BLs: body lateral shape; PFv: pectoral fin vertical position; PFs: Pectoral Fin Size; CPt: caudal peduncle throttling; LGt: longevity, FCt: fecundity; MTg: mature age; TLl: trophic level; TRg temperature range; TMx; maximum temperature; TMn: minimum temperature; EHl: euryhaline; PCr: parental care; DBt: diet breadth.

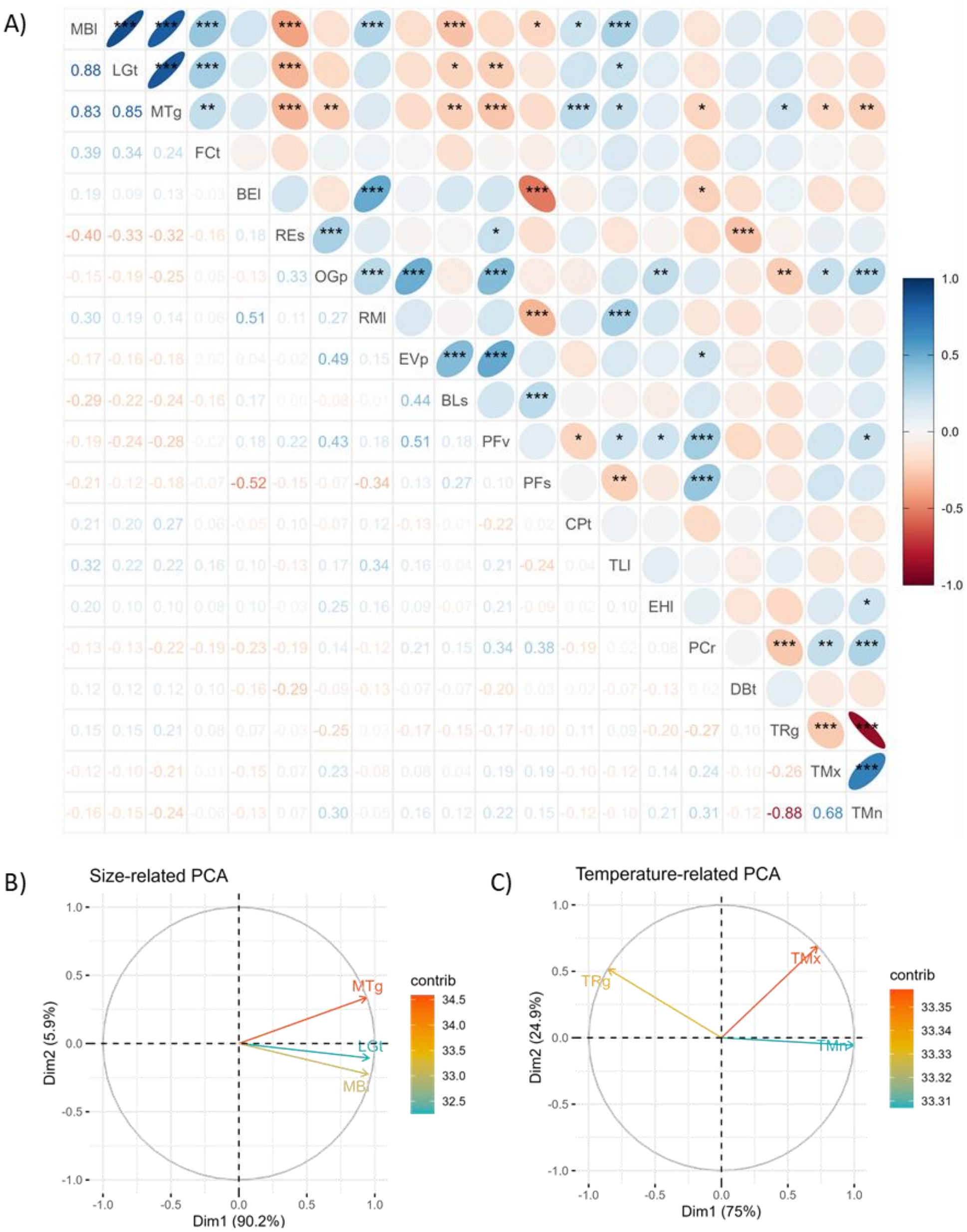

Fig. S8.

Relationship between the 20 functional traits for the 859 fish species in the US. A) Pearson correlation between the traits. B) PCA for the three size-related traits. C) PCA for the three temperature-related traits. MBl: maximum body length; BEl: body elongation; REs: relative eye size; OGp: oral gape position; RMl: relative maxillary length; EVp: eye vertical position; BLs: body lateral shape; PFv: pectoral fin vertical position; PFs: Pectoral Fin Size; CPt: caudal peduncle throttling; LGt: longevity, FCt: fecundity; MTg: mature age; TLl: trophic level; TRg temperature range; TMx; maximum temperature; TMn: minimum temperature; EHl: euryhaline; PCr: parental care; DBt: diet breadth. (two-sides Pearson tests: *** $P < 0.001$, ** $P < 0.01$, * $P < 0.05$; exact P values showed in the lower left panels).

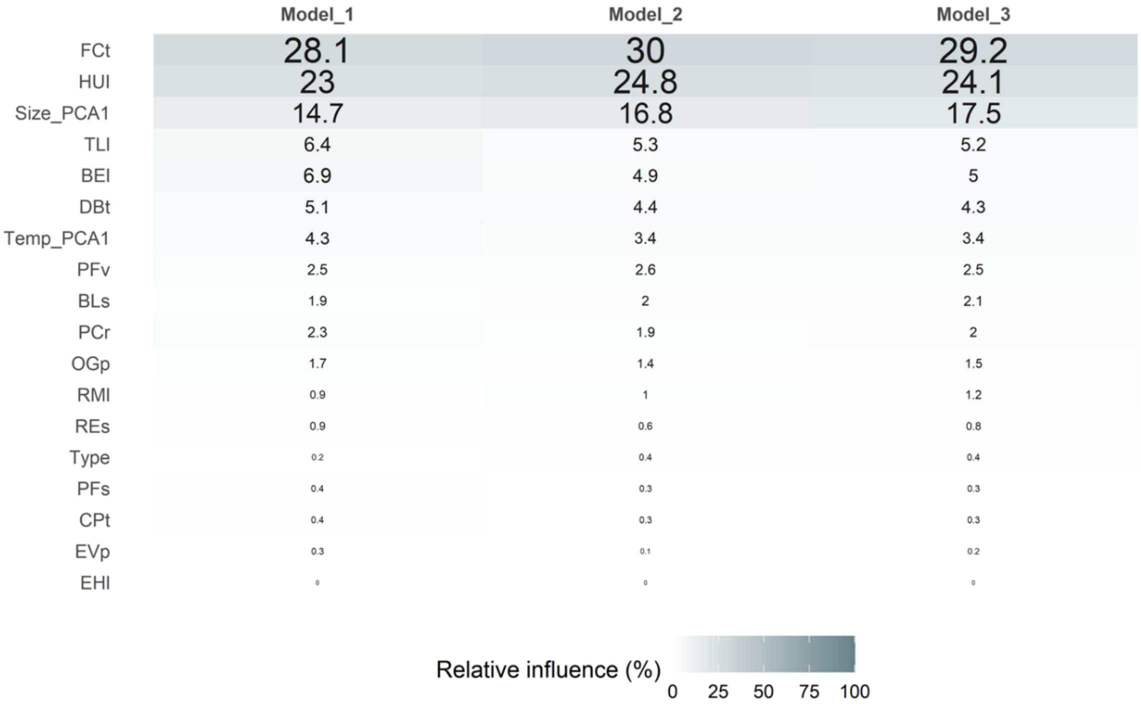

Fig. S9.

Comparation of the relative influence of the 18 predictor variables on the species invasiveness in the three boosted regression tree (BRT) models. Values show the relative influence of each predictor in the models. Model_1: raw data; Model_2: raw data without the seven species with extreme trait values; Model_3: model without the seven species with extreme trait values and with FCt being log_{10}-transformed. FCt: fecundity; HUI: human use index; Size_PCA1: first PC axis of three size-related traits; TLl: trophic level; BEl: body elongation; DBt: diet breadth; Temp_PCA1: first PC axis of three temperature-related traits; BLs: body lateral shape; PFv: pectoral fin vertical position; REs: relative eye size; PCr: parental care; RMl: relative maxillary length; OGp: oral gape position; Type: translated or exotic; CPt: caudal peduncle throttling; EVp: eye vertical position; PFs: Pectoral Fin Size; EHl: euryhaline.

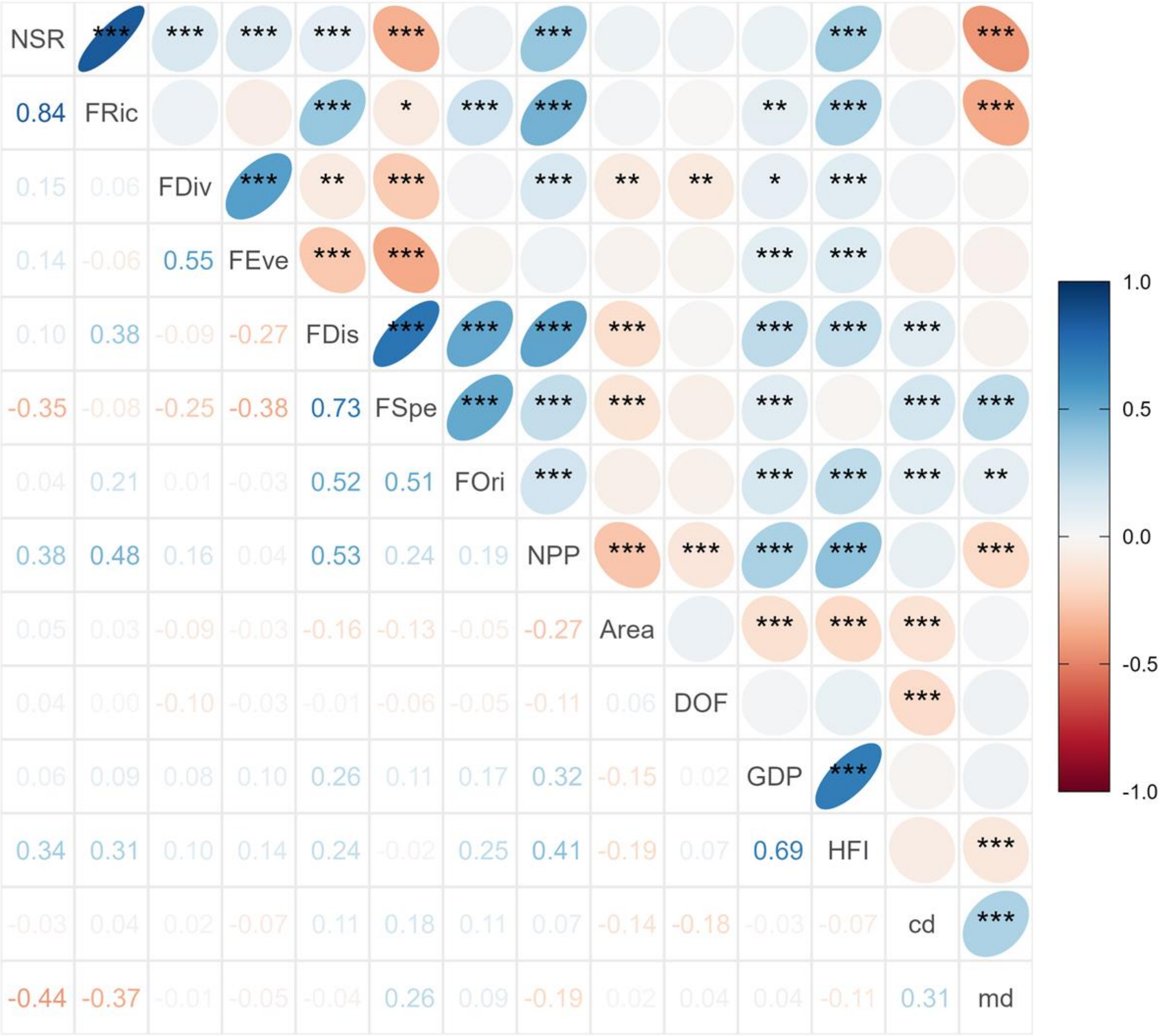

Fig. S10.

Pearson correlation between the 14 predictors considered to evaluate community invasibility (n = 1,868). NSR: native species richness; FRic: functional richness; FDiv: functional divergence; FEve: functional evenness; FDis: functional dispersion; FSpe: functional specialization; FOri: functional originality; DOF: degree of river fragmentation; NPP: net primary productivity; HFI: human footprint index; GDP: gross domestic product; Area: watershed area; cd: centroid distance; md: mean distance. (two-sides Pearson tests: *** $P < 0.001$, ** $P < 0.01$, * $P < 0.05$; exact P values showed in the lower left panels).

Table S1.

Results of Kolmogorov–Smirnov tests for the seventeen quantitative traits and Chi-square tests for the three categorical traits between the native, translocated and exotic species on the 20 functional traits. Bold P-values are significant (P < 0.05). MBl: maximum body length; BEl: body elongation; REs: relative eye size; OGp: oral gape position; RMl: relative maxillary length; EVp: eye vertical position; BLs: body lateral shape; PFv: pectoral fin vertical position; PFs: Pectoral Fin Size; CPt: caudal peduncle throttling; LGt: longevity, FCt: fecundity; MTg: mature age; TLl: trophic level; TRg temperature range; TMx; maximum temperature; TMn: minimum temperature; EHl: euryhaline; PCr: parental care; DBt: diet breadth.

	P-value		
Trait Name	Native-translocated	Native-exotic	Translocated-exotic
MBl	**0.000**	**0.000**	**0.004**
BEl	**0.000**	**0.000**	**0.000**
REs	**0.000**	**0.000**	**0.000**
OGp	**0.004**	**0.022**	0.889
RMl	0.089	**0.006**	**0.020**
EVp	**0.001**	**0.001**	0.366
BLs	**0.000**	**0.000**	**0.040**
PFv	**0.000**	**0.002**	**0.001**
PFs	**0.000**	**0.001**	**0.000**
CPt	**0.001**	0.871	**0.020**
LGt	**0.000**	**0.000**	**0.000**
FCt	**0.000**	**0.000**	0.641
MTg	**0.000**	0.110	0.129
TLl	**0.000**	**0.000**	0.052
TRg	**0.000**	**0.012**	**0.000**
TMx	**0.006**	**0.002**	**0.000**
TMn	**0.000**	**0.002**	**0.000**
EHl	**0.000**	**0.000**	**0.003**
PCr	0.580	**0.000**	**0.000**
DBt	**0.000**	**0.000**	**0.000**

Table S2.

Type and source of the 20 functional traits of fish species in the United States.

Trait Name	Abb.	Data type	Data source
**Morphological**			
Maximum Body Length	MBl	quantitative	Froese & Pauly, 2018
Body Elongation	BEl	quantitative	Brosse et al., 2021
Relative Eye Size	REs	quantitative	Brosse et al., 2021
Oral Gape Position	OGp	quantitative	Brosse et al., 2021
Relative Maxillary Length	RMl	quantitative	Brosse et al., 2021
Eye Vertical Position	EVp	quantitative	Brosse et al., 2021
Body Lateral Shape	BLs	quantitative	Brosse et al.,2021
Pectoral Fin Vertical Position	PFv	quantitative	Brosse et al., 2021
Pectoral Fin Size	PFs	quantitative	Brosse et al., 2021
Caudal Peduncle Throttling	CPt	quantitative	Brosse et al., 2021
**Life-historical**			
Longevity	LGt	quantitative	Frimpong & Angermeier, 2009
Fecundity	FCt	quantitative	Frimpong & Angermeier, 2009
Mature age	MTg	quantitative	Frimpong & Angermeier, 2009
Trophic Level	TLl	quantitative	Froese & Pauly, 2018
Temperature Range	TRg	quantitative	Frimpong & Angermeier, 2009
Maximum Temperature	TMx	quantitative	Frimpong & Angermeier, 2009
Minimum Temperature	TMn	quantitative	Frimpong & Angermeier, 2009
Euryhaline	EHl	binary	Frimpong & Angermeier, 2009
Parental Care	PCr	categorical	Frimpong & Angermeier, 2009
Diet Breadth	DBt	categorical	Frimpong & Angermeier, 2009

Table S3.

Statistical summary of the five environmental and human-related variables in the 1,868 watersheds in US.

**Variables**	**Mean**	**SD**	**Minimum**	**Median**	**Maximum**
Watershed area (Area, km^2)	3628.7	2113.2	89.8	3122.2	22209.1
Net primary productivity (NPP, $gc/m^2/yr$)	5187.1	2423.1	882.3	4847.1	14967.7
Degree of fragmentation (DOF)	7.1	7.4	0.0	5.2	60.7
Gross domestic product (GDP, US$)	18.1	37.6	0.0	6.6	415.4
Human footprint index (HFI)	9.1	6.6	0.0	7.5	39.9

Table S4.

Results of spatially explicit simultaneous autoregressive (SAR) error models for the community invasibility to the established non-native species. Columns show the coefficient estimates (coef), standard error (SE), z-statistic value (z-value) and P-value (two-sided tests) for each variable. The significant P-values (< 0.05) are in bold. The last two rows show the pseudo R-squared values and Moran's I statistic of the SAR model. ns: non-significant.

Variable	coef	SE	z_value	p-value
cd	-2.527	0.381	-6.635	**0.000**
cd2	1.206	0.392	3.080	**0.002**
Area	2.016	0.225	8.964	**0.000**
Area2	-0.768	0.208	-3.687	**0.000**
GDP	1.461	0.327	4.473	**0.000**
GDP2	-1.056	0.245	-4.319	**0.000**
FSpe	2.555	0.954	2.678	**0.007**
FSpe2	-1.487	0.916	-1.623	0.105
NPP	-0.751	0.658	-1.141	0.254
NPP2	0.713	0.621	1.149	0.251
md	2.329	0.412	5.649	**0.000**
md2	-1.429	0.416	-3.437	**0.001**
FDiv	-0.633	0.139	-4.550	**0.000**
FRic	0.451	0.165	2.735	**0.006**
FOri	-0.786	0.168	-4.676	**0.000**
DOF	0.569	0.097	5.901	**0.000**
HFI	1.025	0.178	5.744	**0.000**
FEve	0.279	0.143	1.943	0.052
Pseudo R-suqared	0.51			
Moran's I statistic	-0.0386 (ns)			

Supplementary References

1. Froese, R. & Pauly, D. FishBase. Retrieved from www.fishbase.org (2018).
2. Brosse, S. *et al.* FISHMORPH: A global database on morphological traits of freshwater fishes. *Global Ecology and Biogeography* **30**, 2330– 2336 (2021).
3. Frimpong, E. A. & Angermeier, P. L. Fish traits: a database of ecological and life-history traits of freshwater fishes of the United States. *Fisheries* **34**, 487-495 (2009).
