# Supplementary material for: Species invasiveness and community invasibility of North American freshwater fish fauna revealed via trait-based analysis
Source: Nat Commun. 2023 Apr 22;14:2332. doi: 10.1038/s41467-023-38107-2 (PMC10122662; doi:10.1038/s41467-023-38107-2)
Supplement: Supplementary file 3 — Reporting Summary [file 41467_2023_38107_MOESM3_ESM.pdf]

nature portfolio

Corresponding author(s): Guohuan Su

Last updated by author(s): Apr 7, 2023

Reporting Summary

Nature Portfolio wishes to improve the reproducibility of the work that we publish. This form provides structure for consistency and transparency in reporting. For further information on Nature Portfolio policies, see our Editorial Policies and the Editorial Policy Checklist.

Statistics

For all statistical analyses, confirm that the following items are present in the figure legend, table legend, main text, or Methods section.

n/a	Confirmed	
☐	☒	The exact sample size (*n*) for each experimental group/condition, given as a discrete number and unit of measurement
☒	☐	A statement on whether measurements were taken from distinct samples or whether the same sample was measured repeatedly
☐	☒	The statistical test(s) used AND whether they are one- or two-sided<br>*Only common tests should be described solely by name; describe more complex techniques in the Methods section.*
☐	☒	A description of all covariates tested
☐	☒	A description of any assumptions or corrections, such as tests of normality and adjustment for multiple comparisons
☐	☒	A full description of the statistical parameters including central tendency (e.g. means) or other basic estimates (e.g. regression coefficient) AND variation (e.g. standard deviation) or associated estimates of uncertainty (e.g. confidence intervals)
☐	☒	For null hypothesis testing, the test statistic (e.g. *F*, *t*, *r*) with confidence intervals, effect sizes, degrees of freedom and *P* value noted<br>*Give P values as exact values whenever suitable.*
☒	☐	For Bayesian analysis, information on the choice of priors and Markov chain Monte Carlo settings
☒	☐	For hierarchical and complex designs, identification of the appropriate level for tests and full reporting of outcomes
☐	☒	Estimates of effect sizes (e.g. Cohen's *d*, Pearson's *r*), indicating how they were calculated

Our web collection on statistics for biologists contains articles on many of the points above.

Software and code

Policy information about availability of computer code

Data collection	R language (4.1 version); QGIS (3.18 version)
Data analysis	R language (4.1 version); QGIS (3.18 version); missForest algrithm; custom codes: https://doi.org/10.5281/zenodo.7802871

For manuscripts utilizing custom algorithms or software that are central to the research but not yet described in published literature, software must be made available to editors and reviewers. We strongly encourage code deposition in a community repository (e.g. GitHub). See the Nature Portfolio guidelines for submitting code & software for further information.

Data

Policy information about availability of data

All manuscripts must include a data availability statement. This statement should provide the following information, where applicable:

- Accession codes, unique identifiers, or web links for publicly available datasets
- A description of any restrictions on data availability
- For clinical datasets or third party data, please ensure that the statement adheres to our policy

All data needed to evaluate the conclusions in the paper are present in the paper and/or the Supplementary information. FISHMORPH is publicly available through figshare (https://doi.org/10.6084/m9.figshare.14891412). Fish Traits database for North American freshwater fishes can access through https://www.sciencebase.gov/catalog/item/5a7c6e8ce4b00f54eb2318c0. Additional data and files related to this paper have been deposited in the Zenodo repository (https://doi.org/10.5281/zenodo.7802871)75.

March 2021

Human research participants

Policy information about studies involving human research participants and Sex and Gender in Research.

Reporting on sex and gender	not applicable
Population characteristics	not applicable
Recruitment	not applicable
Ethics oversight	not applicable

Note that full information on the approval of the study protocol must also be provided in the manuscript.

Field-specific reporting

Please select the one below that is the best fit for your research. If you are not sure, read the appropriate sections before making your selection.

☐ Life sciences ☐ Behavioural & social sciences ☒ Ecological, evolutionary & environmental sciences

For a reference copy of the document with all sections, see nature.com/documents/nr-reporting-summary-flat.pdf

Ecological, evolutionary & environmental sciences study design

All studies must disclose on these points even when the disclosure is negative.

Study description	We used trait-based analysis to profile invasive species and quantify the community invasibility for >1,800 North American freshwater fish communities.
Research sample	We collected the US freshwater fish occurrence data from NatureServe and the .S. Geological Survey (USGS) Non-indigenous Aquatic Species (NAS) database. We sampled ish data from the US because US is one of the most fish invaded regions.
Sampling strategy	All data that collected was used in the following analysis, so no sample-size calculation was performed
Data collection	We collected the fish species occurrence data from NatureServe (https://www.natureserve.org) and the U.S. Geological Survey (USGS) Non-indigenous Aquatic Species (NAS) database (http://nas.er.usgs.gov). We collected the fish functional trait data from FISHMORPH database, the Fish Traits database for North American freshwater fishes and FishBase.
Timing and spatial scale	1960s-2010s; 1,868 HUC8 watersheds in the continental US. We collected the data from 1960s to 2010s because most fish introductions and invasions occurred during this periods.
Data exclusions	This occurrence dataset only considered the identified species that locally create self-sustaining populations, thus we excluded records of non-self-sustaining or eradicated populations, vagrant species detected in only one sampling occasion, and non-identified or hybrid species.
Reproducibility	Data and codes for the analysis are available to reproduce all the results.
Randomization	This is not relevant. Species whether are native or invasive species based on their occurrence records status.
Blinding	Blinding was not relevant.

Did the study involve field work? ☐ Yes ☒ No

Reporting for specific materials, systems and methods

We require information from authors about some types of materials, experimental systems and methods used in many studies. Here, indicate whether each material, system or method listed is relevant to your study. If you are not sure if a list item applies to your research, read the appropriate section before selecting a response.

Materials & experimental systems

n/a	Involved in the study
☒	☐ Antibodies
☒	☐ Eukaryotic cell lines
☒	☐ Palaeontology and archaeology
☒	☐ Animals and other organisms
☒	☐ Clinical data
☒	☐ Dual use research of concern

Methods

n/a	Involved in the study
☒	☐ ChIP-seq
☒	☐ Flow cytometry
☒	☐ MRI-based neuroimaging
